# Dealing with missing standard deviation and mean values in meta-analysis of continuous outcomes: a systematic review

**DOI:** 10.1186/s12874-018-0483-0

**Published:** 2018-03-07

**Authors:** Christopher J. Weir, Isabella Butcher, Valentina Assi, Stephanie C. Lewis, Gordon D. Murray, Peter Langhorne, Marian C. Brady

**Affiliations:** 1 Usher Institute of Population Health Sciences and Informatics, University of Edinburgh, Edinburgh, UK; 2 Institute of Cardiovascular and Medical Sciences, University of Glasgow, Glasgow, UK; 3 Nursing, Midwifery and Allied Health Professions Research Unit, Glasgow Caledonian University, Glasgow, UK

**Keywords:** Continuous outcomes, Meta-analysis, Systematic review, Missing mean, Missing standard deviation

## Abstract

**Background:**

Rigorous, informative meta-analyses rely on availability of appropriate summary statistics or individual participant data. For continuous outcomes, especially those with naturally skewed distributions, summary information on the mean or variability often goes unreported. While full reporting of original trial data is the ideal, we sought to identify methods for handling unreported mean or variability summary statistics in meta-analysis.

**Methods:**

We undertook two systematic literature reviews to identify methodological approaches used to deal with missing mean or variability summary statistics. Five electronic databases were searched, in addition to the Cochrane Colloquium abstract books and the Cochrane Statistics Methods Group mailing list archive. We also conducted cited reference searching and emailed topic experts to identify recent methodological developments. Details recorded included the description of the method, the information required to implement the method, any underlying assumptions and whether the method could be readily applied in standard statistical software. We provided a summary description of the methods identified, illustrating selected methods in example meta-analysis scenarios.

**Results:**

For missing standard deviations (SDs), following screening of 503 articles, fifteen methods were identified in addition to those reported in a previous review. These included Bayesian hierarchical modelling at the meta-analysis level; summary statistic level imputation based on observed SD values from other trials in the meta-analysis; a practical approximation based on the range; and algebraic estimation of the SD based on other summary statistics. Following screening of 1124 articles for methods estimating the mean, one approximate Bayesian computation approach and three papers based on alternative summary statistics were identified. Illustrative meta-analyses showed that when replacing a missing SD the approximation using the range minimised loss of precision and generally performed better than omitting trials. When estimating missing means, a formula using the median, lower quartile and upper quartile performed best in preserving the precision of the meta-analysis findings, although in some scenarios, omitting trials gave superior results.

**Conclusions:**

Methods based on summary statistics (minimum, maximum, lower quartile, upper quartile, median) reported in the literature facilitate more comprehensive inclusion of randomised controlled trials with missing mean or variability summary statistics within meta-analyses.

**Electronic supplementary material:**

The online version of this article (10.1186/s12874-018-0483-0) contains supplementary material, which is available to authorized users.

## Background

Systematic review and meta-analysis of clinical trial results is considered the highest level of evidence [1] and provides a readily accessible synthesis of the evidence on the effectiveness of a given treatment. Given the influential role of meta-analysis in shaping clinical guidelines and in turn patient care, it is critical that reviews summarise the available research findings with minimum bias and maximum precision, in order to provide conclusions that are of use to patients and healthcare professionals and that act as an effective guide to future research priorities.

Although reviewers use systematic and transparent methods to minimise bias and random variability in their evaluations of interventions [2], selective or incomplete reporting of trials is problematic [3] and can lead to imprecision and biases in meta-analysis findings.

In the absence of individual participant data, the standard approach to meta-analysis of continuous outcomes requires information on the mean and either the standard deviation (SD), variance or standard error (SE) values for each treatment group [4]. Where continuous outcomes have a skewed distribution, incomplete reporting of the SD and mean is more widespread: often other summaries are reported, with the quartiles or minimum and maximum being given instead of the SD and the median being stated in place of the mean.

In such situations, the systematic reviewer must either exclude the trial from the meta-analysis – with accompanying concerns over the introduction of potential for bias and the loss of precision – or find an alternative method of deriving the missing mean or SD information based on information available in the trial report.

A previous systematic review [5] identified a range of possible methods for replacing missing SD values and concluded that this diversity of approaches implied inconsistency within the systematic review community and suboptimal choice of methods by many. Hozo and colleagues [6] present formulae for estimating the mean value on the basis of other summary statistics and conclude that imputation of the median value is not always adequate, particularly where the sample size is small.

In this paper we update the systematic review of methodology for handling missing SD values and provide a corresponding review of approaches to determining the mean value where this is missing from a clinical trial report. We also consider whether the methods identified could be readily applied by systematic review teams, without recourse to specialist software.

## Methods

### Aims

The first aim of this review was to provide an update on new developments since a previous review [5] of methods, in the meta-analysis context, of determining the variance, SD or SE where these are missing from the original trial report. Methods applicable to parallel group or crossover randomised controlled trials were considered. Our second aim was to provide a systematic review of the methods available to determine the mean value, where this is missing from the original trial report. Finally, we generated illustrative meta-analysis scenarios based on individual participant data from a large completed stroke trial [7] to demonstrate the use of the most readily applicable methods and to compare their performance.

### Overall review strategy

Full details of the data sources for each review topic are given below. Following removal of duplicates and articles published outside the time limits of the review, a single reviewer (IB) screened articles for relevance on the basis of the title and abstract, and then performed a full text review of promising articles to allow formal assessment of eligibility. The full text of articles considered potentially eligible was assessed by an independent reviewer (CJW) to confirm inclusion; any disagreements were resolved via discussion. Pre-specified information from identified papers was recorded jointly by the two reviewers.

Details sought from included articles were the description of the method being proposed, the information required to implement the method and any underlying assumptions. We also considered whether an update to the Cochrane RevMan software [8] would be needed to apply the method and whether it could be readily implemented in other standard statistical software. The findings for the two review topics were summarised descriptively and critically appraised. The search strategy included grey literature to minimise the risk of publication bias.

### Search strategy for missing variance/SD/SE methods

To identify new methods applicable when the variance, SD or SE is missing from the original clinical trial report, we searched the electronic databases MEDLINE, EMBASE, Web of Knowledge, PsycINFO and Global Health. A complex search strategy, developed in consultation with a librarian with bibliographic database searching expertise, was required to provide a specific yet sensitive search; these terms were then tailored to each electronic search engine. We searched for terms such as “derived”, “missing” and “imputed” combined with “variance”, “SD” and “SE”. As an example, the EMBASE search strategy is given in Additional file 1 fig. S1 and illustrates the detail of terms required to obtain a sufficiently specific search which still captures a known key reference [5]. We also searched the grey literature, including Cochrane Colloquium abstract books and the Cochrane Statistics Methods Group mailing list archive. We extended this search to identify recent methodology developments by emailing topic experts on the Cochrane Statistics Methods Group. Full text of articles was obtained from Journals@Ovid (OVFT), YourJournals@Ovid, PsycARTICLES Full Text, Books@Ovid or via inter-library loan.

We included methods belonging to five of the categories identified by Wiebe et al. [5]: 1. Methods to derive the variance/SD/SE algebraically, for example from parametric test statistics or *p*-values; 2. Summary statistic level imputation of the variance/SD/SE (described as “study-level” imputation in Wiebe et al. [5]), for example substituting SD data from other studies, using the coefficient of variation, non-parametric summaries, or correlation data; 3. Meta-analysis level strategies, for example multiple imputation or bootstrapping; 4. Methods to meta-analyse effects on continuous outcomes without using individual study variance/SD/SE; 5. Methods to impute effect size, from which the variance/SD/SE could be derived. Publications from 2002 onwards were included, to ensure complete coverage of the period following that covered by the review of Wiebe et al. [5].

A study was excluded if it presented methods that could not be applied to continuous outcomes or that applied to statistics other than the variance, SD or SE. Papers which applied a technique but did not present new methodology were also excluded.

### Search strategy for missing mean methods

To identify novel methods applicable when the mean is missing from the original clinical trial report, we initially planned to search the same electronic databases, full text and grey literature sources as in the missing variance/SD/SE review. Based on the considerable overlap in that review between hits identified from different electronic sources, we performed a more restricted search using EMBASE and grey literature only, plus a targeted Web of Knowledge search to identify references which cited Hozo et al. [6]. The EMBASE search strategy is given in online Additional file 2 fig. S2. Again, we augmented this search for recent methodology developments by surveying the Cochrane Statistics Methods Group members.

The inclusion and exclusion criteria remained in place from the first review. Publications from 2005 onwards were included: Hozo and colleagues [6] had already summarised previously available methods.

### Method evaluation using individual participant data

Using individual participant data [7] we generated meta-analyses to illustrate the most readily applicable statistical methods for replacing missing variability or mean values. We selected methods which could be implemented without specialist software or statistical programming. We compared the performance of each method to analysis of the complete data (with no missing summary statistics) using

1. its bias (difference between the estimated and true values of the intervention effect), and
2. its imprecision (ratio of the widths of the confidence intervals for the intervention effect [width when estimating missing SD or mean: width when all summary statistics available])

We also compared the performance of each statistical method to the strategy of omitting any trials with missing SD or mean values from the meta-analysis.

We analysed hospital length of stay, an outcome with a skewed distribution for which the SD and mean summary statistics might well be omitted from published trial reports. The intervention effect was the mean difference in days, estimated using random effects meta-analysis fitted in the Cochrane RevMan software v5.3 [8].

The exemplar data set from the carotid surgery randomised trial [7] contains data on 3526 participants from 95 sites in 24 countries. For these illustrative analyses, we selected sites that included 15 or more participants (58 sites; 3268 participants). Meta-analyses scenarios were generated in which each site represented a separate trial, covering various sample sizes and numbers of ‘trials’.

The methods for estimating missing SD or mean values were explored across several meta-analysis scenarios in a fractional factorial design. The factors considered were: (1) number of trials in the meta-analysis (5, 10, 20, 30); (2) trial size: small, large or mixed, containing a mean of 23, 87 and 60 participants per trial respectively; (3) number of trials with missing data (2 for meta-analyses containing 5 trials; 2 and 5 for 10-trial meta-analyses; 5 and 10 for 20-trial meta-analyses; 5, 10 and 15 for 30-trial meta-analyses); and (4) size of the trials with missing summary statistics (small, large or randomly selected).

## Results

In the description of methods, we use the notation: *a*, minimum value; *q1*, lower quartile; *m*, median; *q3*, upper quartile; *b*, maximum; *n*, sample size; and $\overline{x}$, sample mean.

### Missing variance/SD/SE methods

**Fig. 1:**
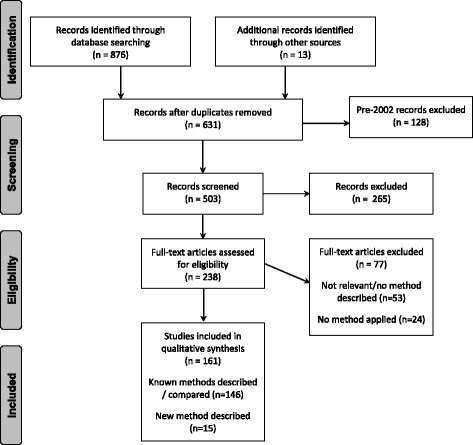
Systematic review of methods to derive missing variance/SD: PRISMA Flow Diagram. *Flow diagram based on:* Moher D, Liberati A, Tetzlaff J, Altman DG, The PRISMA Group (2009). *P*referred *R*eporting *I*tems for *S*ystematic Reviews and *M*eta-*A*nalyses: The PRISMA Statement. PLoS Med 6(6): e1000097. doi:10.1371/journal.pmed1000097

The variance/SD/SE search was run on 12 November 2014 and updated using cited reference searching on 4 April 2016 and the survey of Cochrane topic experts in May 2016. Fig. 1 illustrates the numbers of papers identified, screened, assessed for eligibility and included. Two hundred and thirty-eight were assessed for eligibility following title and abstract screening. Of these, 161 of potential interest were identified; most were previous methodology reviews (2) or systematic review papers (144) which did not present new methodology.

Table 1 summarises details of the articles included. Nine of the 15 methods identified (Abrams et al. [9], Sung et al. [10], Nixon et al. [11], Dakin et al. [12], MacNeil and Graham [13], Stevens [14], Stevens et al. [15], Boucher [16] and Kwon and Reis [17]) are set in a Bayesian framework.

**Table 1:** Summary of methods identified for replacing missing variance/SD/SE

Method	Category	Description	Statistics required	Assumptions	Software implementation
Abrams et al. (2005)	3	Bayesian meta-analysis estimates within-patient correlation between baseline and follow-up; enables imputation of mean change from baseline and its SD when only baseline and follow-up means and SDs reported	• baseline mean/SD • follow-up mean/SD • change from baseline mean/SD in some included studies	SD at baseline same as SD at follow-up; within subject correlation comes from same distribution for all studies and treatment arms; careful choice of prior distribution for variance parameters	Example WinBUGS [24] code provided in paper
Hozo et al. (2005)	1	Missing variance estimated for example as:	• minimum • median • maximum • sample size	Data normally distributed	Excel spreadsheet provided by Wan et al. (2014)
		$$ Var\approx \frac{\left(n+1\right)}{48n{\left(n-1\right)}^2}+\left(\left({n}^2+3\right){\left(a-2m+b\right)}^2+4{n}^2{\left(b-a\right)}^2\right) $$			
Sung et al. (2006)	3	Imputation of missing variances within Bayesian meta-analysis assuming distributed as True variance * χ^2^ (n-1)/(n-1) where true variance distributed as log-normal	• variances reported for other included studies	Assume missing variances come from same lognormal distribution as reported variances	Implemented in WinBUGS; code supplied in online supplement to article
Walter and Yao (2007)	1	Improved version of “range” method which calculates SD = (b-a)/4	• sample size • range or min/max	Approximate normality	Lookup table in paper could readily be implemented in standard software; RevMan [8] could accommodate in update
Ma et al. (2008)	2	Impute weighted average of variances observed in other studies; or calculate a range of pooled estimates for efficacy based on the smallest and largest variances observed	• sample size • variances of other studies in meta-analysis	Unobserved and observed variances come from the same underlying distribution	Could readily be implemented in any statistical software
Nixon et al. (2009)	3	Impute missing change from baseline SD values in Bayesian random effects meta-regression	• baseline SD • follow-up SD	Log transform of baseline SD, follow-up SD and change from baseline SD follow trivariate normal distribution. Where follow-up SD is based on complete cases, imputation assumes non-informative drop-out	Applied in WinBUGS
Dakin et al. (2010)	3	Bayesian hierarchical modelling estimating SD values in context of network meta-analysis. SD assumed to follow gamma distribution; parameters estimated from studies reporting SDs	• observed SDs	Observed and missing SD values come from the same gamma distribution	WinBUGS code provided in publication
MacNeil et al. (2010)	3	Impute missing SDs in hierarchical Bayesian meta-analysis based on posterior predictive distribution	• observed SDs	Observed, missing SDs arise from same gamma distribution	Implemented in PyMC Markov chain Monte Carlo (MCMC) toolkit [31] of Python [32]; code given in online supplement
Stevens (2011), Stevens et al. (2012)	3	Bayesian network meta-analysis that enables imputation of missing SDs via posterior predictive distribution (variances assumed to follow gamma distribution)	• observed variances	Variances follow gamma distribution; log(SD) given weak uniform prior distribution	WinBUGS code provided
Boucher (2012)	3	Emax model of SDs; implemented using either maximum likelihood or hierarchical Bayesian model	• observed SDs over time in longitudinal study	longitudinal modelling of SDs using Emax mixed effects model; differences by treatment group permitted in SDs; weak uniform prior for SD used in Bayesian approach	SAS (SAS Institute Inc., Cary, NC) PROC NLMIXED and WinBUGS code provided for maximum likelihood and Bayesian approaches respectively
Wan et al. (2014)*	1	$SD\approx \frac{q3-q1}{2{\Phi}^{-1}\left(\frac{0.75n-0.125}{n+0.25}\right)}$	• lower quartile • upper quartile • sample size	Data normally distributed	Excel spreadsheet provided by Wan et al. (2014)
Bland (2015)	1	Missing variance estimated as:	• minimum • lower quartile • median • upper quartile • maximum • sample mean	Data normally distributed	Excel spreadsheet provided by Wan et al. (2014)
		$$ Var\approx \frac{1}{n-1}\left(\frac{\left[2\left(\mathrm{n}+3\right)\left(\mathrm{q}12+\mathrm{m}2+\mathrm{q}32\right)+2\left(\mathrm{n}-5\right)\left(\mathrm{a}.\mathrm{q}1+\mathrm{m}.\mathrm{q}1+\mathrm{m}.\mathrm{q}3+\mathrm{q}3.\mathrm{b}\right)+\left(\mathrm{n}+11\right)\left(\mathrm{a}2+\mathrm{b}2\right)\right]}{16}-\mathrm{n}{\overline{x}}^2\right) $$			
Kwon and Reis (2015)	1	Approximate Bayesian computation to estimate SD	• available summary statistics	Underlying distribution of data	R code provided
Chowdhry et al. (2016)	2	Meta-regression assuming sample variances follow gamma distribution	• observed variances from other studies in meta-analysis • study covariates	Variances missing at random (MAR) and follow a gamma distribution	Can be fitted in SAS PROC NLMIXED

*a* minimum value, *q1* lower quartile, *m* median, *q3* upper quartile, *b* maximum, *n* sample size, $\overline{x}$, sample mean

*Also provide formulae for scenarios where only a, b and n are available; or where a, b, q1, q3, and n are available; see Results section for details

Key to category numbers:

1 Methods to derive the variance/SD/SE algebraically from parametric test statistics, *p*-values, etc

2 Summary statistic level imputation of variance/SD/SE, for example substituting SD data from other studies, using coefficient of variation, non-parametric summaries, or correlation data

3 Meta-analysis level strategies, for example multiple imputation or bootstrapping

4 Methods to meta-analyse effects on continuous outcomes without using individual study variance/SD/SE

5 Methods to impute effect size, from which variance/SD/SE could be derived

### Category 1. Methods to derive SD/SE/variance algebraically

Walter and Yao [18] present a readily applicable improvement to a method based on the minimum and maximum observed values of the outcome. This “range” method, whereby the difference between minimum and maximum values is divided by 4 to estimate the SD, was originally presented by Mendehall and colleagues [19] in the survey sampling context. In this update, a lookup table of conversion factors from range to SD, based on the distributional results of Tippett [20] for the range, is presented for a variety of sample sizes. They illustrate the method in two example studies of interventions to improve adherence to randomised treatment in rheumatoid arthritis and human immunodeficiency virus. They caution that non-Normality of outcomes, whether through skewness or kurtosis, may invalidate their tabulated conversion factors but note that the very presence of skewness might be the cause of the minimum and maximum being reported instead of the SD. They observe that although in theory the use of certain interior order statistics may perform better on skewed outcomes than their proposed method, in practice such statistics would never be available in trial reports; they conclude that their method offers an acceptable compromise in the absence of the original data being obtainable from the original trial publication authors.

Hozo and colleagues [6] present a formula (see Table 1) for estimating the variance where values for the minimum, median, maximum and sample size are available. In simulations of outcomes from a range of parametric distributions, they find that their approximation performs best on Normally distributed data when the sample size is very small but methods based on dividing the range by 4 and 6 are superior for sample sizes from 16 to 70 and over 70 respectively. Similar patterns are observed on skewed outcomes simulated from log-Normal, beta, exponential and Weibull distributions. In simulated meta-analyses, they conclude that their variance estimation formula may miss the true value by a margin between 10% and 20%.

Bland [21] presents a formula for the variance (Table 1) which makes use of the lower quartile and upper quartile in addition to the minimum, median and maximum. In simulations, Bland demonstrates that his formula overestimates the standard deviation at larger sample sizes where the underlying distribution is Normal; the issue is exacerbated for skewed outcomes. Nevertheless, in both situations the formula provides a less biased estimate than that of Hozo et al. [6]. The over-estimation is attributed to the greater chance of extreme outliers occurring in large sample sizes, thus inflating the estimation of the variance through the minimum and maximum values. He considers that the method will still be useful, since studies in meta-analyses with a small sample size are most likely to be the ones with unreported SD values and source data that cannot be obtained from the trial report authors.

Continuing the theme of estimation based on summary statistics, Wan et al. [22], due to concerns over the restrictive non-negative data assumption and the arbitrary sample size thresholds guiding choice of formula in the method of Hozo and colleagues [6], propose an improvement using the same summary statistics:

$$ SD\approx \frac{b-a}{2{\Phi}^{-1}\left(\frac{n-0.375}{n+0.25}\right)} $$

and an enhancement to the approach of Bland [21] that additionally takes account of sample size with the aim of reducing overestimation at larger sample sizes:

$$ SD\approx \frac{b-a}{4{\Phi}^{-1}\left(\frac{n-0.375}{n+0.25}\right)}+\frac{q3-q1}{4{\Phi}^{-1}\left(\frac{0.75n-0.125}{n+0.25}\right)} $$

Finally, using only the lower quartile, upper quartile and sample size, they propose the following estimate:

$$ SD\approx \frac{q3-q1}{2{\Phi}^{-1}\left(\frac{0.75n-0.125}{n+0.25}\right)} $$

and note its similarity to the Cochrane Handbook [4] estimator

$$ SD\approx \frac{q3-q1}{1.35} $$

Through simulations, they demonstrate superior estimation properties, for both Normal and skewed data, of their respective extensions to the methods of Hozo and Bland. They also illustrate that a valid estimate of the SD may also be made when the minimum and maximum are unavailable but the upper and lower quartiles are reported.

Kwon and Reis [17] apply simulation-based approximate Bayesian computation (ABC) in estimating missing SD values based on other summary statistics available in the trial report. The likelihood function for Bayesian inference is unlikely to be evaluable, due to the unavailability of all data points from the source trials in a meta-analysis. They therefore propose ABC, which replaces the likelihood by using a distance measure – in this example the Euclidean distance – to compare summary statistics between the observed and simulated data. The prior distribution for the outcome must be specified: they propose that the underlying probability distribution (for example, Normal or log-Normal) may be determined based on background knowledge of the outcome, and a uniform prior should be placed on each parameter of this distribution, informed by the available summary statistics. Many sets of candidate parameter values are then generated from this prior, and from these, many pseudo-data sets. Each of the pseudo data sets is then compared to the observed summary statistics and accepted if these are sufficiently close (for example in the top 0.1% smallest Euclidean distances). This accepted set of parameter values is then used to estimate the parameter of interest, in this case the SD. In simulations of outcomes from Normal and skewed distributions, they find the ABC method performs consistently better for skewed distributions than the formulae of Hozo, Bland and Wan. ABC does not perform as well when the sample size is less than about 40 and the method of Wan et al. [22] is superior for Normally distributed outcomes.

### Category 2. Summary statistic level imputation

Ma et al. [23] present two summary statistic level imputation methods which make use of the variances observed in other trials in the meta-analysis. In the first (termed the “prognostic” method) they calculate the average of the observed variances and use this in the study with missing variance information. In the second (the “interval method”) they calculate a range of pooled estimates for efficacy by imputing, for trials with missing variances, the minimum and maximum of the variances observed in other trials in the meta-analysis. They illustrate the methods in meta-analyses of drugs for patients with type 2 diabetes and interventions to lower intra-ocular pressure in open angle glaucoma or intraocular hypertension. They conclude that the prognostic method is preferable and also gives more stable results than the policy of omitting trials with missing variance summary statistics from a meta-analysis. They caution that if the trials with observed variances are small, the resulting imprecision in these estimates will lead to poor performance of the prognostic method.

### Category 3. Meta-analysis level strategies

Abrams et al. [9] accommodate differences in methodology among included trials (for example, where outcome is reported at a given follow-up point rather than as a change from baseline). This can lead to missing information on, for example, the SD of mean change from baseline. Their proposed solution (in contrast to single imputation methods or omission of studies not reporting change from baseline summary statistics) is to adopt a fully Bayesian approach in which external information is used to build a prior distribution for the within-patient correlation between baseline and follow-up measures, thus enabling appropriate estimation of the SD of the change from baseline where only the baseline, and follow-up SD values have been reported. A Uniform(0,1) vague prior for the correlation ρ is used. Where external evidence is available, this prior is replaced by performing a Bayesian meta-analysis of the Fisher transformations *S*_*j*_ of the observed ρ_j_ from external studies *j* = 1,…,J; a vague Normal prior with mean δ is placed on the *S*_*j*_ and the back-transformation $\uprho =\frac{e^{2\delta }-1}{e^{2\delta }+1}$ is used. They conclude that such an approach gives a substantial improvement to meta-analysis estimation of the pooled mean difference compared to applying a fixed value for ρ. They note the conclusions are sensitive to the choice of prior distribution, particularly when a limited number of studies is included in the meta-analysis, and that study-level covariates may be included to incorporate greater flexibility in the prior for ρ.

Sung et al. [10] incorporate continuous outcomes with missing variances in a Bayesian meta-analysis by estimating the distribution of reported variances and applying multiple imputation of missing variances, assuming that these arose from this “parent” log-Normal distribution. Specifically, the missing variance is assumed to be distributed as the true variance, multiplied by a χ^2^ distribution divided by its degrees of freedom. The degrees of freedom equal n-1, where n is the sample size of the trial with unreported variance. This is a special case of a gamma distribution, with shape (n-1)/2 and scale (n-1)/(2 * true variance). Sung et al. contend that their approach offers advantages, in comparison to discarding information from studies with unreported variances, and consider it more straightforward to implement in a Bayesian framework using the WinBUGS software than it would be to employ a frequentist equivalent.

In a systematic review of valsartan in the treatment of hypertension, Nixon et al. [11] impute missing SD values within a Bayesian random effects meta-regression. The missing data imputation model assumes a trivariate normal distribution for the log-transformed baseline SD, follow-up SD and change from baseline SD. The following relationship between the SD measures is exploited: ${S}_{di}^2={S}_{1i}^2+{S}_{2i}^2-2{\rho}_{12}{S}_{1i}{S}_{2i}$, where *S*_*di*_ , *S*_1*i*_ and *S*_2*i*_ are the change from baseline SD, baseline SD and follow-up SD respectively, and ρ_12_ is the within-patient correlation between baseline and follow-up. The variance of observed SDs is weighted by the inverse of the sample size. The meta-regression allows adjustment for study-level characteristics, such as mean baseline value, which may influence the treatment effect. As with other imputation approaches identified in this review, the missing at random assumption applies.

Dakin et al. [12], in the context of a mixed treatment comparison meta-analysis, perform Bayesian modelling of SDs contained in trial reports. They estimate the gamma distribution that these follow and sample values from that distribution to impute the SD for studies in which this is missing, while still enabling the uncertainty around these imputed values to be taken into account in the meta-analysis. The unreported SD values are assumed to be missing completely at random.

Within the setting of a hierarchical Bayesian meta-analysis of the biogeographical relationship between coral reef loss and populations of fish that rely on coral, MacNeil and Graham [13] impute missing standard deviations from their posterior predictive distribution based on the observed SD data. Again, any missing SDs are assumed missing completely at random and the uncertainty in imputed values is retained in the subsequent hierarchical meta-analysis.

In the framework of a network meta-analysis, Stevens [14] generates the posterior predictive distribution of missing variances via Markov Chain Monte Carlo using WinBUGS [24], assuming a gamma distribution for the observed variances. The log-transformed SD values are given a weak uniform prior. Using an example data set where the true study and treatment group specific SDs are known, he illustrates that the assumption of a common standard deviation (missing completely at random) may not be tenable and that violation of this leads to problems in pooled treatment effect estimation. He further highlights the importance of examining the role of study-specific covariates in predicting the observed SDs. Stevens et al. [15] implement the same technique in a network meta-analysis of treatments for intermittent claudication.

Boucher [16] imputes missing variances using a non-linear mixed effects Emax model of SDs over time in the specific scenario where longitudinal measurements of a pain outcome are available but not all SDs are reported. The SD for study *i,* treatment group *j*, time point *k* is modelled as

$$ {SD}_{ijk}={E}_0+\frac{\left({E}_{\mathit{\max}0}\ast \left(1-{I}_j\right)+{E}_{\mathit{\max}1}\ast {I}_j\right)}{et_{50}+{t}_{ijk}}+{\eta}_i+{\xi}_{ijk} $$

where *E*_0_ is the estimated baseline SD, *E*_*max*0_ and *E*_*max*1_ are the maximum difference over baseline for treatment groups 0 and 1 respectively, *I*_*j*_ is an indicator variable for treatment group, *et*_50_ is the time post first dose when 50% of the maximal difference over baseline is reached, *t*_*ijk*_ is the time post first dose, ${\eta}_i\sim \mathrm{N}\left(0,{\sigma}_{bsd}^2\right)$ is the between study variability, and ${\xi}_{ijk}\sim \mathrm{N}\left(0,{\sigma}_{sd}^2/{n}_{ijk}\right)$ is the residual. Maximum likelihood and Bayesian approaches to estimation are investigated. Weak priors are used so that only the observed SDs inform the missing data imputation. A joint model encompassing missing SD imputation and the final meta-analysis ensures that uncertainty in the imputed values is carried forward to the meta-analysis. He concludes that a Bayesian modelling approach holds advantages (in terms of appropriate propagation of uncertainty) over maximum likelihood techniques. Either approach would require unreported SDs to be missing completely at random.

Chowdhry et al. [25] impute missing variances for meta-analyses of parallel group and cross-over trials using a gamma meta-regression generalised linear mixed model, additionally taking study covariates into account when modelling the variance. The study random effect reflects the reasonable assumption that the between-study variation in variance cannot entirely be explained by the available covariates. They perform inference on the mean treatment difference using multiple imputation. The method depends on the missing at random (MAR) assumption regarding unreported variances. They propose sensitivity analyses via a pattern mixture model if variances are missing not at random (MNAR). The approach may benefit from a large number of trials being included in the meta-analysis: their motivating example covers 84 parallel group trials. Their extensive simulation studies demonstrate the superior performance of the method with regard to Type I error and coverage, in comparison to single imputation approaches such as that of Ma et al. [23] or the complete case approach (found in 9% of meta-analyses by Wiebe et al. [5] in which trials with missing variances are omitted from meta-analysis. They conclude that the advantages are smaller than expected, primarily because the missing variances influence only the weighting applied in the meta-analysis.

### Missing mean methods

**Fig. 2:**
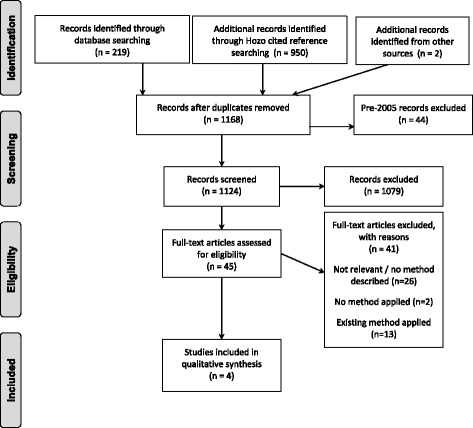
Systematic review of methods to derive missing mean: PRISMA Flow Diagram. *Flow diagram based on:* Moher D, Liberati A, Tetzlaff J, Altman DG, The PRISMA Group (2009). *P*referred *R*eporting *I*tems for *S*ystematic Reviews and *M*eta-*A*nalyses: The PRISMA Statement. PLoS Med 6(6): e1000097. 10.1371/journal.pmed1000097

The search was run on 12 November 2014, and updated using the cited reference search of Hozo and colleagues [6] on 4 April 2016 and the survey of Cochrane topic experts in May 2016. Fig. 2 shows the numbers of papers identified, screened, assessed for eligibility and included. Following title and abstract screening 44 papers were selected for full text review, of which 29 were systematic reviews and 15 were methodology papers. From these, 24 papers discussing or implementing methodology to replace missing mean values were identified. A diversity of approaches was considered but only three [17, 21, 22] presented novel methodology for estimation of a missing mean.

### Category 1. Methods to derive mean algebraically

Hozo and colleagues [6] present methods for deriving a missing mean value where data are available on the median, minimum, maximum and sample size:

$$ \overline{\mathrm{x}}\approx \frac{a+2m+b}{4}+\frac{a-2m+b}{4n} $$

They note that where n is large, the right-hand term in the equation becomes negligibly small and may be omitted. In simulations they confirm that for Normally-distributed data, the formula closely estimates the true mean (within 4% across all scenarios studied), although for larger sample sizes the median was a more accurate estimator. For skewed data, the counter-intuitive result is that the median is a better estimator of the mean for larger samples (about 25 or more), despite the above formula incorporating additional information on the minimum, maximum and sample size.

Bland [21] takes account of the extended scenario where information on the lower (q1) and upper (q3) quartiles is also available:

$$ \overline{\mathrm{x}}\approx \frac{\left(n+3\right)a+2\left(n-1\right)\left(q1+m+q3\right)m+\left(n+3\right)b}{8n} $$

Bland additionally notes that such quantile based methods readily apply even if log-transformation of an outcome seems appropriate: the log-transformed quantiles may be used in the above formula to estimate the mean of the log-transformed outcome. This is of particular interest in the case of skewed data, where unreported mean values are more likely. Simulation studies based on Normally-distributed data show that the mean estimation formula of Bland shows minimal bias. For skewed data, the mean estimation approach shows somewhat less than half the bias found in the method of Hozo and colleagues [6]. Bland observes that in meta-analysis, the interest will often be in the difference between treatment group means and any bias in mean estimation would therefore cancel out as it would be present in both groups.

Wan et al. [22] provide a further method which applies in situations where the lower (q1) and upper (q3) quartiles are available but the minimum and maximum are not.

$$ \overline{\mathrm{x}}\approx \frac{q1+m+q3}{3} $$

They also provide simplified versions of the equations provided by Hozo et al. [6] and Bland [21], as well as an Excel spreadsheet to aid practical implementation which contains all of their formulae as well as those of Hozo et al. [6] and Bland [21]. Simulations show the Wan et al. [22] formula estimates the mean unbiasedly in the case of Normally-distributed data; for skewed data according to the log-Normal, Beta, Weibull or Exponential distributions, at larger sample sizes (greater than *n* = 100) it provides a smaller relative error in the mean estimation than the approach of Bland, even though it does not include the minimum and maximum summary statistics.

Kwon and Reis [17] also apply the simulation-based approximate Bayesian computation (ABC) approach described in the missing variance/SD/SE section to estimate missing mean values. In simulation studies they find that for a sample size above 40 ABC performs consistently better than the methods of Hozo et al.[6], Bland [21] and Wan et al. [22] across all scenarios; its benefit is greatest where the underlying continuous outcome distribution is skewed or heavy-tailed. The average relative error of ABC estimation of the mean is almost zero for sample sizes over 100. The ABC approach does however require the underlying probability distribution (for example, log-Normal) to be specified in advance; the performance of the method under model misspecification was not investigated.

### Category 2. Summary statistic level imputation

As also observed in the review by Wiebe et al. [5], many studies applied sensitivity analysis to evaluate the impact of imputation strategies. For example, Fedorowicz et al. [26] planned to implement best and worst case scenarios (worst observed trial mean for intervention and best trial mean for comparator; and vice versa) as sensitivity analysis imputation strategies, citing the Cochrane Handbook [4] guidance on dealing with missing data.

### Method evaluation using individual participant data

We implemented illustrative meta-analyses, selecting the subset of methods identified in the systematic reviews that we considered most readily applicable by systematic reviewers without the requirement for specialist software or programming skills. For handling missing SD/SE/variance summaries we selected the single imputation approach of Ma and colleagues [23], the look-up table method of Walter and Yao [18] and the Cochrane Handbook [4] formula (which takes a very similar form to that of Wan et al. [22]). For dealing with missing mean values, we applied the formula of Hozo and colleagues [6] as an important reference method; in addition, we implemented the algebraic recalculations presented by Bland [21] and Wan et al. [22].

Table 2 shows the results of the illustrative analyses for missing SD/SE/variance, for the selected methods versus the comparators of (1) complete data set analysis and (2) omitting trials with missing data. There was little difference across methods in terms of bias in the estimated mean difference, which was at most 0.23 days in magnitude and varied little across meta-analysis scenarios. In contrast, the imprecision in the estimate of the mean treatment effect increased substantially with the proportion of trials with missing SDs (for example the confidence interval width increased by a factor of 2.19 when 15 of 30 trials had a missing SD and those trials were omitted from the meta-analysis). The method of Walter and Yao gave greatest protection against this increased imprecision, performing better than all alternative methods in every scenario. Its imprecision was at most 1.17, in cases where 15 of 30 trials had a missing SD.

**Table 2:** GALA results: missing SD

			Method for missing SD replacement							
	Complete data result		Ma [23]		Walter [18]		Cochrane Handbook [4]		None, omit study	
	Mean difference (days)	95% confidence interval	Bias	Imprecision	Bias	Imprecision	Bias	Imprecision	Bias	Imprecision
**Scenario**										
**5 trials**	− 0.01	(− 0.87, 0.85)								
2 missing SD			0.21	1.18	0.05	1.05	−0.01	0.73	0.27	1.28
**10 trials**	−0.01	(− 0.37, 0.35)								
2 missing SD			0.04	1.04	−0.01	1.01	−0.02	1.97	0.02	1.06
5 missing SD			−0.03	1.56	0.01	1.10	−0.12	2.40	0.00	1.64
**20 trials**	0.00	(−0.31, 0.30)								
5 missing SD			0.07	1.26	0.02	1.02	0.06	2.74	0.02	1.25
10 missing SD			0.26	2.20	0.02	1.07	0.17	3.93	0.05	1.41
**30 trials**	−0.01	(−0.28, 0.25)								
5 missing SD			0.06	1.11	0.03	1.06	−0.12	2.23	0.09	1.15
10 missing SD			−0.09	1.49	−0.01	1.13	−0.21	2.45	−0.02	1.62
15 missing SD			−0.03	1.87	0.02	1.17	−0.23	2.28	0.12	2.19

Results are given for mixed sample size scenario (average of 60 patients per trial) and random allocation of trials to missing SD values. Imprecision is the ratio of the widths of the confidence intervals for the intervention effect [width when estimating missing SDs: width when all SDs available]. Results for other scenarios (small trials, large trials; missing SD in small trials, large trials) show similar patterns and are available in online Additional file 3 Tables S1-S8

Table 3 gives the findings for the missing mean illustrative meta-analyses in a similar format. In general bias was low, with the exception of the Hozo method which showed notable bias in meta-analyses containing 20 trials. The Wan formula exhibited minimal imprecision across all scenarios, outperforming all other methods. The exception was for 5-trial meta-analyses with missing means for two trials, where the Hozo and Bland methods also demonstrated negligible imprecision; however in this case the Wan approach showed lower bias in the estimated treatment effect.

**Table 3:** GALA results: missing mean

			Method for missing mean replacement							
	Complete data result		Hozo [6]		Bland [21]		Wan [22]		None, omit study	
	Mean difference (days)	95% confidence interval	Bias	Imprecision	Bias	Imprecision	Bias	Imprecision	Bias	Imprecision
**Scenario**										
**5 trials**	−0.01	(−0.87, 0.85)								
2 missing means			−0.20	1.00	−0.12	1.00	0.05	1.00	0.27	1.28
**10 trials**	−0.01	(− 0.37, 0.35)								
2 missing means			0.64	3.47	0.15	2.15	−0.03	1.00	0.02	1.06
5 missing means			0.12	4.35	−0.06	1.58	0.04	1.00	0.00	1.64
**20 trials**	0.00	(−0.31, 0.30)								
5 missing means			1.16	3.67	0.38	2.30	−0.03	1.00	0.02	1.25
10 missing means			1.02	4.34	0.34	2.66	0.01	1.00	0.05	1.41
**30 trials**	−0.01	(−0.28, 0.25)								
5 missing means			0.02	1.43	0.01	1.15	0.01	1.00	0.09	1.15
10 missing means			0.01	2.92	0.06	1.89	0.04	1.00	−0.02	1.62
15 missing means			−0.19	3.26	−0.05	2.13	0.03	1.00	0.12	2.19

Results are given for mixed sample size scenario (average of 60 patients per trial) and random allocation of trials to missing mean values. Imprecision is the ratio of widths of confidence intervals for the intervention effect [width when estimating missing means: width when all means available] Results for other scenarios (small trials, large trials; missing mean in small trials, large trials) show similar patterns (online Additional file 3 Tables S9-S16)

## Discussion

These parallel reviews provide an update on current methodological approaches to the inclusion of studies identified during the course of a systematic review that have a missing mean or variability summary statistic in meta-analysis of a continuous outcome. For missing SD values, fifteen new methods were identified in addition to those summarised by Wiebe and colleagues [5]. Methods identified for estimating the mean were the approximate Bayesian computation approach of Kwon and Reis [17] and formulae from Wan et al. [22], Bland [21] and Hozo and colleagues [6] based on specific sets of summary statistics. Neither review identified any new methods to meta-analyse effects on continuous outcomes that do not use individual study mean or variance/SD/SE; or new methods to impute effect size, from which the mean or variance/SD/SE could be derived.

The recent literature includes a substantial number of methods in addition to those for missing variance/SD/SE reviewed by Wiebe and colleagues [5] and the methods for estimating the mean available at the time of the seminal publication by Hozo and colleagues [6]. Nevertheless, we support the sequence of steps proposed in the Wiebe et al. review that systematic reviewers should follow to handle missing trial summary data, with one notable modification. The original sequence was (1) use algebraic recalculation to recover missing summaries; (2) contact study authors to retrieve the summaries; (3) use multiple imputation if sufficiently many studies with complete information have been included; (4) use non-parametric summaries if these have been reported and the distribution of the outcome is not markedly skewed; (5) use single imputation if at least one sufficiently similar study is included in the review; (6) summarise non-pooled data from studies with missing summaries alongside the meta-analysis of studies without missing summaries; and (7) perform weighted meta-analytic tests, avoiding mean or SD summaries and instead making use of available *p*-value and sample size information. We add a further recommendation: after step (2), that approximate algebraic calculations such as those of Hozo, Bland and Wan should be attempted if study authors have not provided the required data. Our rationale is that approximate algebraic approaches will be readily implementable by the vast majority of systematic reviewers, whereas step (3) requires specialist statistical expertise and subsequent steps require further assumptions or do not fully incorporate the study missing a summary statistic in the meta-analysis.

The Bayesian methodologies identified in the review contributed to two areas. First, approximate Bayesian computing was implemented to estimate the missing summary statistic [17]. ABC enables estimation when the full likelihood function cannot be enumerated, as is often the case in meta-analysis, where the data available are study level summaries rather than individual level data. Secondly, Bayesian strategies contributed all of the new meta-analysis level methods identified in our reviews [9, 10, 11, 12, 13, 14, 15, 16, 17]. In general these approaches utilised Bayesian hierarchical modelling to represent the meta-analysis data structure. Missing SD or mean values were either imputed following assignment of a vague prior distribution or were estimated by sampling from an empirical prior drawn from studies which did report the relevant summary statistic.

We highlight three main considerations for systematic reviewers when selecting a method for handling missing mean or variability summaries: applicability; assumptions; and availability of the required data.

### Applicability

While Bayesian hierarchical modelling and approximate Bayesian computation offer much flexibility for systematic review groups in which this expertise is present, these methods are not currently available in standard meta-analysis software and therefore remain unavailable to the majority of systematic reviewers. In contrast, the approximate algebraic approaches of Hozo [6], Bland [21] and Wan [22] are readily applied in Excel and statistical software. The single imputation approach of Ma et al. [23] and the look-up table in Walter and Yao [18] are also reasonably accessible. Ease of use of a particular method must of course be balanced against its assumptions, so for example sensitivity to the assumption of Normality inherent in approximate algebraic approaches would require careful evaluation.

### Assumptions

Many of the methods described in this review assume that data are Normally distributed. Skewness, or other non-Normal features, may be the underlying reason for an SD or mean being absent from a clinical trial report and so any method will need to be robust to deviations from Normality. Encouragingly, despite having theoretical dependence on Normality, many methods performed well when estimating the mean or SD from data with log-Normal, Weibull, Exponential or Beta distributions.

When mean or SD data are missing in a trial report, it is essential to investigate possible mechanisms that would have led to this, and to use this knowledge when choosing an imputation method [27]. Almost all of the identified methods in this review assumed that the relevant summary statistics would be missing completely at random or at best, missing at random. The plausibility of such assumptions will often be questionable, and so conducting appropriate sensitivity analyses to explore the assumptions of the selected imputation method will also form an essential part of the process. The strategy which omits studies with missing summary statistics from the meta-analysis should be one such sensitivity analysis, given its relatively strong performance across our illustrative meta-analysis scenarios.

### Availability of data

In the event of a mean or SD being unreported for a clinical trial, there is also no guarantee that summary statistics required by the methods identified in this review would be available. We therefore caution against advocating a single approach. Several methods could be applied within a single meta-analysis, on a trial-by trial basis, depending on which statistics were available for each trial. The ease of applicability and strong estimation performance of the Walter and Yao method for missing SDs cannot be utilised if the minimum and maximum values (or range) of the data are unreported. Similarly, the straightforward method of Wan estimates the mean well but requires information on the median and lower and upper quartiles. For example, in a systematic review of early-supported discharge interventions following acute stroke [28], the minimum and maximum of the hospital length of stay outcome were reported in four of eight original trial publications (50%) and its median, lower and upper quartiles were reported for two of eight trials (25%). This emphasises the importance of having a suite of methods available to handle unreported summary statistics.

For continuous outcomes which have a highly skewed distribution, it remains an open question whether basing a meta-analysis on the mean treatment effect is the most appropriate approach. Future research should investigate alternative approaches not based on the mean, for example building on the work of Higgins and colleagues [29] which enables results from the original and log-transformed scales to be combined in a meta-analysis to provide a geometric mean summary of treatment effect. When a meta-analysis of a highly skewed outcome is based on mean and SD summary statistics, the implicit assumption is that the systematic review will have identified enough sufficiently large trials that the central limit theorem would apply. We note that this will not universally be the case in all areas of medicine or for the full range of research questions addressed by meta-analysis.

Our review had a number of strengths and limitations. Because of the frequent usage of the search terms of interest (mean, standard deviation) in manuscript text it was a challenge to develop a sufficiently specific electronic search which was also sensitive. This, and the pattern of duplicate hits across electronic database sources in the SD methods search, led us to narrow the scope of the electronic searching for methodology to handle missing mean values to the EMBASE database only. We offset this limitation by performing cited reference searching on known key papers and by surveying topic experts from the Cochrane Statistics Methods Group. This also provided assurance that our review findings reflect the current state of the art, given the time gap from our original electronic searching to reporting. Our use of multiple sources (electronic, grey literature, expert opinion) provides protection against publication bias. The lack of a substantial treatment effect on the length of stay outcome in our illustrative meta-analyses using GALA trial [7] data may have limited the extent to which the potential bias of the selected methods could be evaluated. By presenting the mean and SD/SE/variance reviews together, we provide a single resource for systematic reviewers seeking guidance on the issue.

## Conclusions

Ultimately, full reporting of the original trial data is preferable [30] to any efforts to recover missing mean or SD values. Nevertheless, the range of methods identified in this review remain important as they allow systematic reviewers performing meta-analysis to incorporate as much information as possible from completed trials which have already reported, often giving an advantage over the omission of trials with missing mean or SD values from a meta-analysis.

## Additional files

Additional file 1: **Fig. S1.** Example EMBASE search for methods to replace missing variance, SD or standard error. (DOCX 20 kb)

Additional file 2: **Fig. S2.** Example EMBASE search for methods to replace missing mean. (DOCX 20 kb)

Additional file 3: **Tables S1-S8.** GALA results: missing SD. **Tables S9-S16.** GALA results: missing mean. (DOCX 83 kb)

## Abbreviations

ABC: Approximate Bayesian Computation
SD: Standard deviation
SE: Standard error
