# Supplementary material for: Dealing with missing standard deviation and mean values in meta-analysis of continuous outcomes: a systematic review
Source: BMC Med Res Methodol. 2018 Mar 7;18:25. doi: 10.1186/s12874-018-0483-0 (PMC5842611; doi:10.1186/s12874-018-0483-0)
Supplement: Supplementary file 2 — Fig. S2. Example EMBASE search for methods to replace missing mean. (DOCX 20 kb) [file 12874_2018_483_MOESM2_ESM.docx]

1. exp "meta analysis (topic)"/ or Meta-Analysis/ or exp Review Literature as Topic/ or Review Literature.mp.

2. (meta-analy$ or metaanaly$ or (meta adj analy$) or metanaly$).tw.

3. (systematic adj5 (review$1 or overview$1)).tw. or systematic review/

4. clinical trial/ or controlled clinical trial/ or Clinical Trials as Topic/ or (clinical adj3 trial$1).tw. or controlled clinical trial.mp.

5. randomized controlled trial/ or "randomized controlled trial (topic)"/ or (randomi#ed adj5 trial$1).tw.

6. *data analysis/ or *data extraction/ or *data synthesis/

7. *statistics as topic/ or *statistical parameters/ or *variance/ or *statistical analysis/ or *"analysis of covariance"/ or *"analysis of variance"/ or *attributable risk/ or *bootstrapping/ or *canonical analysis/ or *chi square test/ or *cohort analysis/ or *correlation analysis/ or *correspondence analysis/ or *effect size/ or *etiologic fraction/ or *fisher exact test/ or *frequency analysis/ or *friedman test/ or *geostatistical analysis/ or *inferential statistics/ or *instrumental variable analysis/ or *intention to treat analysis/ or *jackknife test/ or *kaplan meier method/ or *kappa statistics/ or *kolmogorov smirnov test/ or *kruskal wallis test/ or *latent structure analysis/ or *life table method/ or *log rank test/ or *loglinear model/ or *mantel haenszel test/ or *maximum likelihood method/ or *mcnemar test/ or *median test/ or *meta analysis/ or *"meta analysis (topic)"/ or *monte carlo method/ or *most probable number method/ or *multilevel analysis/ or *multivariate analysis/ or *nonparametric test/ or *numbers needed to treat/ or *one tailed test/ or *ordination analysis/ or *parametric test/ or *post hoc analysis/ or *power analysis/ or *"power of a test"/ or *principal coordinate analysis/ or *rank sum test/ or *rasch analysis/ or *redundancy analysis/ or *regression analysis/ or *risk benefit analysis/ or *sequential analysis/ or *sign test/ or *spatial analysis/ or *spatial autocorrelation analysis/ or *student t test/ or *temporal analysis/ or *two tailed test/ or *univariate analysis/ or *wilcoxon signed ranks test/ or *yates continuity correction/ or *youden index/

8. exp *statistical parameters/

9. (data adj5 (pool or pooled or pooling$)).tw.

10. 1 or 2 or 3 or 4 or 5 or 6 or 7 or 8 or 9

11. (imput* adj2 mean$1).tw.

12. (missing adj mean$1).tw.

13. (derive* adj mean$1).tw.

14. ((extracte* adj2 mean$1) not "extracted by").tw.

15. (MISSING adj3 OUTCOME).tw.

16. 11 or 12 or 13 or 14 or 15

17. 10 and 16
