# Supplementary material for: Dealing with missing standard deviation and mean values in meta-analysis of continuous outcomes: a systematic review
Source: BMC Med Res Methodol. 2018 Mar 7;18:25. doi: 10.1186/s12874-018-0483-0 (PMC5842611; doi:10.1186/s12874-018-0483-0)
Supplement: Supplementary file 3 — Tables S1-S8. GALA results: missing SD. Tables S9-S16. GALA results: missing mean. (DOCX 83 kb) [file 12874_2018_483_MOESM3_ESM.docx]

**Table S1 GALA results: missing SD**

|  |  |  | **Method for missing SD replacement** | | | | | | | |
| --- | --- | --- | --- | --- | --- | --- | --- | --- | --- | --- |
|  | **Complete data result** | | **Ma (ref)** | | **Walter (ref)** | | **Cochrane Handbook** | | **None, omit study** | |
|  | **Mean difference (days)** | **95% confidence interval** | **Bias** | **Imprecision** | **Bias** | **Imprecision** | **Bias** | **Imprecision** | **Bias** | **Imprecision** |
| **Scenario** |  |  |  |  |  |  |  |  |  |  |
| **5 trials** | -0.01 | (-0.87, 0.85) |  |  |  |  |  |  |  |  |
| 2 missing SD |  | | 0.18 | 1.28 | 0.01 | 1.03 | 0.10 | 0.80 | 0.26 | 1.58 |
| **10 trials** | -0.01 | (-0.37, 0.35) |  |  |  |  |  |  |  |  |
| 2 missing SD |  | | -0.16 | 1.38 | -0.03 | 1.06 | -0.17 | 1.31 | -0.16 | 1.39 |
| 5 missing SD |  | | -0.08 | 2.31 | -0.01 | 1.14 | -0.32 | 1.61 | -0.03 | 2.75 |
| **20 trials** | 0.00 | (-0.31, 0.30) |  |  |  |  |  |  |  |  |
| 5 missing SD |  | | -0.12 | 1.25 | -0.02 | 1.10 | -0.25 | 3.02 | -0.11 | 1.44 |
| 10 missing SD |  | | -0.19 | 1.64 | -0.02 | 1.11 | -0.23 | 2.92 | -0.23 | 1.75 |
| **30 trials** | -0.01 | (-0.28, 0.25) |  |  |  |  |  |  |  |  |
| 5 missing SD |  | | -0.02 | 1.00 | -0.01 | 1.00 | -0.01 | 1.00 | -0.06 | 1.15 |
| 10 missing SD |  | | -0.11 | 1.21 | -0.02 | 1.04 | 0.09 | 2.81 | -0.14 | 1.28 |
| 15 missing SD |  | | -0.07 | 1.49 | -0.01 | 1.09 | 0.07 | 2.64 | -0.10 | 1.60 |

Results are given for mixed sample size scenario (average of 60 patients per trial) and allocation of the smallest trials to have missing SD values. Imprecision is the ratio of the widths of the confidence intervals for the intervention effect [width when estimating missing SDs: width when all SDs available].

**Table S2 GALA results: missing SD**

|  |  |  | **Method for missing SD replacement** | | | | | | | |
| --- | --- | --- | --- | --- | --- | --- | --- | --- | --- | --- |
|  | **Complete data result** | | **Ma (ref)** | | **Walter (ref)** | | **Cochrane Handbook** | | **None, omit study** | |
|  | **Mean difference (days)** | **95% confidence interval** | **Bias** | **Imprecision** | **Bias** | **Imprecision** | **Bias** | **Imprecision** | **Bias** | **Imprecision** |
| **Scenario** |  |  |  |  |  |  |  |  |  |  |
| **5 trials** | -0.01 | (-0.87, 0.85) |  |  |  |  |  |  |  |  |
| 2 missing SD |  | | -0.15 | 1.44 | -0.10 | 1.13 | 0.29 | 1.30 | -0.20 | 1.53 |
| **10 trials** | -0.01 | (-0.37, 0.35) |  |  |  |  |  |  |  |  |
| 2 missing SD |  | | 0.04 | 1.03 | 0.04 | 1.01 | -0.18 | 1.15 | 0.08 | 1.06 |
| 5 missing SD |  | | 0.31 | 2.13 | 0.00 | 1.04 | 0.54 | 2.46 | 0.01 | 1.08 |
| **20 trials** | 0.00 | (-0.31, 0.30) |  |  |  |  |  |  |  |  |
| 5 missing SD |  | | 0.03 | 1.46 | 0.05 | 1.03 | 0.07 | 3.07 | 0.12 | 1.08 |
| 10 missing SD |  | | 0.29 | 2.03 | 0.03 | 1.05 | 0.36 | 2.56 | 0.10 | 1.51 |
| **30 trials** | -0.01 | (-0.28, 0.25) |  |  |  |  |  |  |  |  |
| 5 missing SD |  | | 0.22 | 1.91 | 0.03 | 1.02 | 0.24 | 3.02 | 0.06 | 1.04 |
| 10 missing SD |  | | 0.22 | 1.98 | -0.01 | 1.08 | 0.22 | 2.74 | 0.00 | 1.19 |
| 15 missing SD |  | | 0.40 | 2.38 | 0.01 | 1.11 | 0.41 | 2.57 | 0.07 | 1.30 |

Results are given for mixed sample size scenario (average of 60 patients per trial) and allocation of the largest trials to have missing SD values. Imprecision is the ratio of the widths of the confidence intervals for the intervention effect [width when estimating missing SDs: width when all SDs available].

**Table S3 GALA results: missing SD**

|  |  |  | **Method for missing SD replacement** | | | | | | | |
| --- | --- | --- | --- | --- | --- | --- | --- | --- | --- | --- |
|  | **Complete data result** | | **Ma (ref)** | | **Walter (ref)** | | **Cochrane Handbook** | | **None, omit study** | |
|  | **Mean difference (days)** | **95% confidence interval** | **Bias** | **Imprecision** | **Bias** | **Imprecision** | **Bias** | **Imprecision** | **Bias** | **Imprecision** |
| **Scenario** |  |  |  |  |  |  |  |  |  |  |
| **5 trials** | 2.48 | (-0.12 , 5.08) |  |  |  |  |  |  |  |  |
| 2 missing SD |  | | -2.31 | 0.42 | -2.48 | 0.34 | -2.39 | 0.27 | -2.23 | 0.52 |
| **10 trials** | 0.90 | (-0.25 , 2.05) |  |  |  |  |  |  |  |  |
| 2 missing SD |  | | -1.07 | 0.43 | -0.94 | 0.33 | -1.08 | 0.41 | -1.07 | 0.43 |
| 5 missing SD |  | | -0.99 | 0.72 | -0.92 | 0.36 | -1.23 | 0.50 | -0.94 | 0.86 |
| **20 trials** | 0.19 | (-0.23 , 0.61) |  |  |  |  |  |  |  |  |
| 5 missing SD |  | | -0.31 | 0.90 | -0.21 | 0.80 | -0.44 | 2.19 | -0.30 | 1.05 |
| 10 missing SD |  | | -0.38 | 1.19 | -0.21 | 0.81 | -0.42 | 2.12 | -0.42 | 1.27 |
| **30 trials** | 0.02 | (-0.33 , 0.37) |  |  |  |  |  |  |  |  |
| 5 missing SD |  | | -0.05 | 0.76 | -0.04 | 0.76 | -0.04 | 0.76 | -0.09 | 0.87 |
| 10 missing SD |  | | -0.14 | 0.91 | -0.05 | 0.79 | 0.06 | 2.13 | -0.17 | 0.97 |
| 15 missing SD |  | | -0.10 | 1.13 | -0.04 | 0.83 | 0.04 | 2.00 | -0.13 | 1.21 |

Results are given for trials with smaller sample sizes (average of 23 patients per trial) and allocation of the smallest trials to have missing SD values. Imprecision is the ratio of the widths of the confidence intervals for the intervention effect [width when estimating missing SDs: width when all SDs available].

**Table S4 GALA results: missing SD**

|  |  |  | **Method for missing SD replacement** | | | | | | | |
| --- | --- | --- | --- | --- | --- | --- | --- | --- | --- | --- |
|  | **Complete data result** | | **Ma (ref)** | | **Walter (ref)** | | **Cochrane Handbook** | | **None, omit study** | |
|  | **Mean difference (days)** | **95% confidence interval** | **Bias** | **Imprecision** | **Bias** | **Imprecision** | **Bias** | **Imprecision** | **Bias** | **Imprecision** |
| **Scenario** |  |  |  |  |  |  |  |  |  |  |
| **5 trials** | 2.48 | (-0.12 , 5.08) |  |  |  |  |  |  |  |  |
| 2 missing SD |  | | -2.28 | 0.39 | -2.44 | 0.35 | -2.50 | 0.24 | -2.22 | 0.42 |
| **10 trials** | 0.90 | (-0.25 , 2.05) |  |  |  |  |  |  |  |  |
| 2 missing SD |  | | -0.87 | 0.33 | -0.92 | 0.32 | -0.93 | 0.62 | -0.89 | 0.33 |
| 5 missing SD |  | | -0.94 | 0.49 | -0.90 | 0.34 | -1.03 | 0.75 | -0.91 | 0.51 |
| **20 trials** | 0.19 | (-0.23 , 0.61) |  |  |  |  |  |  |  |  |
| 5 missing SD |  | | -0.12 | 0.92 | -0.17 | 0.74 | -0.13 | 1.99 | -0.17 | 0.90 |
| 10 missing SD |  | | 0.07 | 1.60 | -0.17 | 0.77 | -0.02 | 2.86 | -0.14 | 1.02 |
| **30 trials** | 0.02 | (-0.33 , 0.37) |  |  |  |  |  |  |  |  |
| 5 missing SD |  | | 0.03 | 0.84 | 0.00 | 0.80 | -0.15 | 1.69 | 0.06 | 0.87 |
| 10 missing SD |  | | -0.12 | 1.13 | -0.04 | 0.86 | -0.24 | 1.86 | -0.05 | 1.23 |
| 15 missing SD |  | | -0.06 | 1.41 | -0.01 | 0.89 | -0.26 | 1.73 | 0.09 | 1.66 |

Results are given for trials with smaller sample sizes (average of 23 patients per trial) and random allocation trials to have missing SD values. Imprecision is the ratio of the widths of the confidence intervals for the intervention effect [width when estimating missing SDs: width when all SDs available].

**Table S5 GALA results: missing SD**

|  |  |  | **Method for missing SD replacement** | | | | | | | |
| --- | --- | --- | --- | --- | --- | --- | --- | --- | --- | --- |
|  | **Complete data result** | | **Ma (ref)** | | **Walter (ref)** | | **Cochrane Handbook** | | **None, omit study** | |
|  | **Mean difference (days)** | **95% confidence interval** | **Bias** | **Imprecision** | **Bias** | **Imprecision** | **Bias** | **Imprecision** | **Bias** | **Imprecision** |
| **Scenario** |  |  |  |  |  |  |  |  |  |  |
| **5 trials** | 2.48 | (-0.12 , 5.08) |  |  |  |  |  |  |  |  |
| 2 missing SD |  | | -2.64 | 0.48 | -2.59 | 0.37 | -2.20 | 0.43 | -2.69 | 0.51 |
| **10 trials** | 0.90 | (-0.25 , 2.05) |  |  |  |  |  |  |  |  |
| 2 missing SD |  | | -0.87 | 0.32 | -0.87 | 0.32 | -1.09 | 0.36 | -0.83 | 0.33 |
| 5 missing SD |  | | -0.60 | 0.67 | -0.91 | 0.33 | -0.37 | 0.77 | -0.90 | 0.34 |
| **20 trials** | 0.19 | (-0.23 , 0.61) |  |  |  |  |  |  |  |  |
| 5 missing SD |  | | -0.16 | 1.06 | -0.14 | 0.75 | -0.12 | 2.23 | -0.07 | 0.79 |
| 10 missing SD |  | | 0.10 | 1.48 | -0.16 | 0.76 | 0.17 | 1.86 | -0.09 | 1.10 |
| **30 trials** | 0.02 | (-0.33 , 0.37) |  |  |  |  |  |  |  |  |
| 5 missing SD |  | | 0.19 | 1.44 | 0.00 | 0.77 | 0.21 | 2.29 | 0.03 | 0.79 |
| 10 missing SD |  | | 0.19 | 1.50 | -0.04 | 0.81 | 0.19 | 2.07 | -0.03 | 0.90 |
| 15 missing SD |  | | 0.37 | 1.80 | -0.02 | 0.84 | 0.38 | 1.94 | 0.04 | 0.99 |

Results are given for trials with smaller sample sizes (average of 23 patients per trial) and allocation of the largest trials to have missing SD values. Imprecision is the ratio of the widths of the confidence intervals for the intervention effect [width when estimating missing SDs: width when all SDs available].

**Table S6 GALA results: missing SD**

|  |  |  | **Method for missing SD replacement** | | | | | | | |
| --- | --- | --- | --- | --- | --- | --- | --- | --- | --- | --- |
|  | **Complete data result** | | **Ma (ref)** | | **Walter (ref)** | | **Cochrane Handbook** | | **None, omit study** | |
|  | **Mean difference (days)** | **95% confidence interval** | **Bias** | **Imprecision** | **Bias** | **Imprecision** | **Bias** | **Imprecision** | **Bias** | **Imprecision** |
| **Scenario** |  |  |  |  |  |  |  |  |  |  |
| **5 trials** | -0.09 | (-1.06, 0.87) |  |  |  |  |  |  |  |  |
| 2 missing SD |  | | 0.26 | 1.14 | 0.09 | 0.92 | 0.18 | 0.72 | 0.34 | 1.41 |
| **10 trials** | -0.31 | (-0.99 , 0.38) |  |  |  |  |  |  |  |  |
| 2 missing SD |  | | 0.14 | 0.72 | 0.27 | 0.55 | 0.13 | 0.69 | 0.14 | 0.73 |
| 5 missing SD |  | | 0.22 | 1.21 | 0.29 | 0.60 | -0.02 | 0.85 | 0.27 | 1.45 |
| **20 trials** | -0.04 | (-0.42 , 0.35) |  |  |  |  |  |  |  |  |
| 5 missing SD |  | | -0.08 | 0.99 | 0.02 | 0.87 | -0.21 | 2.39 | -0.07 | 1.14 |
| 10 missing SD |  | | -0.15 | 1.30 | 0.02 | 0.88 | -0.19 | 2.31 | -0.19 | 1.39 |
| **30 trials** | -0.12 | (-0.42 , 0.18) |  |  |  |  |  |  |  |  |
| 5 missing SD |  | | 0.09 | 0.88 | 0.10 | 0.88 | 0.10 | 0.88 | 0.05 | 1.02 |
| 10 missing SD |  | | 0.00 | 1.07 | 0.09 | 0.92 | 0.20 | 2.48 | -0.03 | 1.13 |
| 15 missing SD |  | | 0.04 | 1.32 | 0.10 | 0.97 | 0.18 | 2.33 | 0.01 | 1.42 |

Results are given for trials with larger sample sizes (average of 87 patients per trial) and selection of the smallest trials to have missing SD values. Imprecision is the ratio of the widths of the confidence intervals for the intervention effect [width when estimating missing SDs: width when all SDs available].

**Table S7 GALA results: missing SD**

|  |  |  | **Method for missing SD replacement** | | | | | | | |
| --- | --- | --- | --- | --- | --- | --- | --- | --- | --- | --- |
|  | **Complete data result** | | **Ma (ref)** | | **Walter (ref)** | | **Cochrane Handbook** | | **None, omit study** | |
|  | **Mean difference (days)** | **95% confidence interval** | **Bias** | **Imprecision** | **Bias** | **Imprecision** | **Bias** | **Imprecision** | **Bias** | **Imprecision** |
| **Scenario** |  |  |  |  |  |  |  |  |  |  |
| **5 trials** | -0.09 | (-1.06, 0.87) |  |  |  |  |  |  |  |  |
| 2 missing SD |  | | 0.29 | 1.05 | 0.13 | 0.94 | 0.07 | 0.65 | 0.35 | 1.14 |
| **10 trials** | -0.31 | (-0.99 , 0.38) |  |  |  |  |  |  |  |  |
| 2 missing SD |  | | 0.34 | 0.55 | 0.29 | 0.53 | 0.28 | 1.04 | 0.32 | 0.55 |
| 5 missing SD |  | | 0.27 | 0.82 | 0.31 | 0.58 | 0.18 | 1.26 | 0.30 | 0.86 |
| **20 trials** | -0.04 | (-0.42 , 0.35) |  |  |  |  |  |  |  |  |
| 5 missing SD |  | | 0.11 | 1.00 | 0.06 | 0.81 | 0.10 | 2.17 | 0.06 | 0.99 |
| 10 missing SD |  | | 0.30 | 1.74 | 0.06 | 0.84 | 0.21 | 3.12 | 0.09 | 1.12 |
| **30 trials** | -0.12 | (-0.42 , 0.18) |  |  |  |  |  |  |  |  |
| 5 missing SD |  | | 0.17 | 0.98 | 0.14 | 0.93 | -0.01 | 1.97 | 0.20 | 1.02 |
| 10 missing SD |  | | 0.02 | 1.32 | 0.10 | 1.00 | -0.10 | 2.17 | 0.09 | 1.43 |
| 15 missing SD |  | | 0.08 | 1.65 | 0.13 | 1.03 | -0.12 | 2.02 | 0.23 | 1.93 |

Results are given for trials with larger sample sizes (average of 87 patients per trial) and random selection of trials to have missing SD values. Imprecision is the ratio of the widths of the confidence intervals for the intervention effect [width when estimating missing SDs: width when all SDs available].

**Table S8 GALA results: missing SD**

|  |  |  | **Method for missing SD replacement** | | | | | | | |
| --- | --- | --- | --- | --- | --- | --- | --- | --- | --- | --- |
|  | **Complete data result** | | **Ma (ref)** | | **Walter (ref)** | | **Cochrane Handbook** | | **None, omit study** | |
|  | **Mean difference (days)** | **95% confidence interval** | **Bias** | **Imprecision** | **Bias** | **Imprecision** | **Bias** | **Imprecision** | **Bias** | **Imprecision** |
| **Scenario** |  |  |  |  |  |  |  |  |  |  |
| **5 trials** | -0.09 | (-1.06, 0.87) |  |  |  |  |  |  |  |  |
| 2 missing SD |  | | -0.07 | 1.28 | -0.02 | 1.01 | 0.37 | 1.16 | -0.12 | 1.36 |
| **10 trials** | -0.31 | (-0.99 , 0.38) |  |  |  |  |  |  |  |  |
| 2 missing SD |  | | 0.34 | 0.54 | 0.34 | 0.53 | 0.12 | 0.61 | 0.38 | 0.55 |
| 5 missing SD |  | | 0.61 | 1.12 | 0.30 | 0.55 | 0.84 | 1.29 | 0.31 | 0.57 |
| **20 trials** | -0.04 | (-0.42 , 0.35) |  |  |  |  |  |  |  |  |
| 5 missing SD |  | | 0.07 | 1.16 | 0.09 | 0.82 | 0.11 | 2.43 | 0.16 | 0.86 |
| 10 missing SD |  | | 0.33 | 1.61 | 0.07 | 0.83 | 0.40 | 2.03 | 0.14 | 1.19 |
| **30 trials** | -0.12 | (-0.42 , 0.18) |  |  |  |  |  |  |  |  |
| 5 missing SD |  | | 0.33 | 1.68 | 0.14 | 0.90 | 0.35 | 2.67 | 0.17 | 0.92 |
| 10 missing SD |  | | 0.33 | 1.75 | 0.10 | 0.95 | 0.33 | 2.42 | 0.11 | 1.05 |
| 15 missing SD |  | | 0.51 | 2.10 | 0.12 | 0.98 | 0.52 | 2.27 | 0.18 | 1.15 |

Results are given for trials with larger sample sizes (average of 87 patients per trial) and selection of the largest trials to have missing SD values. Imprecision is the ratio of the widths of the confidence intervals for the intervention effect [width when estimating missing SDs: width when all SDs available].

**Table S9 GALA results: missing mean**

|  |  |  | **Method for missing mean replacement** | | | | | | | |
| --- | --- | --- | --- | --- | --- | --- | --- | --- | --- | --- |
|  | **Complete data result** | | **Hozo (ref)** | | **Bland (ref)** | | **Wan (ref)** | | **None, omit study** | |
|  | **Mean difference (days)** | **95% confidence interval** | **Bias** | **Imprecision** | **Bias** | **Imprecision** | **Bias** | **Imprecision** | **Bias** | **Imprecision** |
| **Scenario** |  |  |  |  |  |  |  |  |  |  |
| **5 trials** | -0.01 | (-0.87, 0.85) |  |  |  |  |  |  |  |  |
| 2 missing means |  | | -0.11 | 1.00 | -0.09 | 1.00 | -0.16 | 1.00 | 0.26 | 1.58 |
| **10 trials** | -0.01 | (-0.37, 0.35) |  |  |  |  |  |  |  |  |
| 2 missing means |  | | -0.01 | 1.00 | 0.05 | 1.00 | 0.01 | 1.00 | -0.16 | 1.39 |
| 5 missing means |  | | 0.02 | 1.29 | 0.04 | 1.22 | 0.04 | 1.00 | -0.03 | 2.75 |
| **20 trials** | 0.00 | (-0.31, 0.30) |  |  |  |  |  |  |  |  |
| 5 missing means |  | | 0.03 | 1.79 | 0.01 | 1.46 | -0.01 | 0.98 | -0.11 | 1.44 |
| 10 missing means |  | | 0.09 | 1.93 | 0.04 | 1.56 | 0.00 | 1.00 | -0.23 | 1.75 |
| **30 trials** | -0.01 | (-0.28, 0.25) |  |  |  |  |  |  |  |  |
| 5 missing means |  | | 0.05 | 1.28 | 0.01 | 1.09 | -0.03 | 1.00 | -0.06 | 1.15 |
| 10 missing means |  | | 0.03 | 1.62 | 0.00 | 1.28 | -0.02 | 1.00 | -0.14 | 1.28 |
| 15 missing means |  | | 0.04 | 1.91 | 0.01 | 1.45 | 0.03 | 1.00 | -0.10 | 1.60 |

Results are given for mixed sample size scenario (average of 60 patients per trial) and allocation of the smallest trials to have missing mean values. Imprecision is the ratio of widths of confidence intervals for the intervention effect [width when estimating missing means : width when all means available]

**Table S10 GALA results: missing mean**

|  |  |  | **Method for missing mean replacement** | | | | | | | |
| --- | --- | --- | --- | --- | --- | --- | --- | --- | --- | --- |
|  | **Complete data result** | | **Hozo (ref)** | | **Bland (ref)** | | **Wan (ref)** | | **None, omit study** | |
|  | **Mean difference (days)** | **95% confidence interval** | **Bias** | **Imprecision** | **Bias** | **Imprecision** | **Bias** | **Imprecision** | **Bias** | **Imprecision** |
| **Scenario** |  |  |  |  |  |  |  |  |  |  |
| **5 trials** | -0.01 | (-0.87, 0.85) |  |  |  |  |  |  |  |  |
| 2 missing means |  | | 2.02 | 4.78 | 1.02 | 2.79 | -0.20 | 1.00 | -0.20 | 1.53 |
| **10 trials** | -0.01 | (-0.37, 0.35) |  |  |  |  |  |  |  |  |
| 2 missing means |  | | -0.62 | 2.88 | -0.36 | 1.96 | 0.02 | 0.99 | 0.08 | 1.06 |
| 5 missing means |  | | 1.15 | 5.03 | 0.25 | 3.04 | -0.04 | 0.99 | 0.01 | 1.08 |
| **20 trials** | 0.00 | (-0.31, 0.30) |  |  |  |  |  |  |  |  |
| 5 missing means |  | | 0.49 | 4.31 | 0.11 | 2.62 | 0.02 | 1.00 | 0.12 | 1.08 |
| 10 missing means |  | | 1.45 | 5.03 | 0.45 | 3.00 | -0.06 | 0.98 | 0.10 | 1.51 |
| **30 trials** | -0.01 | (-0.28, 0.25) |  |  |  |  |  |  |  |  |
| 5 missing means |  | | 2.98 | 6.58 | 0.92 | 3.68 | 0.01 | 1.00 | 0.06 | 1.04 |
| 10 missing means |  | | 3.44 | 7.00 | 1.14 | 3.91 | -0.03 | 1.00 | 0.00 | 1.19 |
| 15 missing means |  | | 3.98 | 7.11 | 1.29 | 3.98 | -0.07 | 1.00 | 0.07 | 1.30 |

Results are given for mixed sample size scenario (average of 60 patients per trial) and allocation of the largest trials to have missing mean values. Imprecision is the ratio of widths of confidence intervals for the intervention effect [width when estimating missing means : width when all means available]

**Table S11 GALA results: missing mean**

|  |  |  | **Method for missing mean replacement** | | | | | | | |
| --- | --- | --- | --- | --- | --- | --- | --- | --- | --- | --- |
|  | **Complete data result** | | **Hozo (ref)** | | **Bland (ref)** | | **Wan (ref)** | | **None, omit study** | |
|  | **Mean difference (days)** | **95% confidence interval** | **Bias** | **Imprecision** | **Bias** | **Imprecision** | **Bias** | **Imprecision** | **Bias** | **Imprecision** |
| **Scenario** |  |  |  |  |  |  |  |  |  |  |
| **5 trials** | 2.48 | (-0.12 , 5.08) |  |  |  |  |  |  |  |  |
| 2 missing means |  | | -2.60 | 0.33 | -2.58 | 0.33 | -2.65 | 0.33 | -2.23 | 0.52 |
| **10 trials** | 0.90 | (-0.25 , 2.05) |  |  |  |  |  |  |  |  |
| 2 missing means |  | | -0.92 | 0.31 | -0.86 | 0.31 | -0.90 | 0.31 | -1.07 | 0.43 |
| 5 missing means |  | | -0.89 | 0.40 | -0.87 | 0.38 | -0.87 | 0.31 | -0.94 | 0.86 |
| **20 trials** | 0.19 | (-0.23 , 0.61) |  |  |  |  |  |  |  |  |
| 5 missing means |  | | -0.16 | 1.30 | -0.18 | 1.06 | -0.20 | 0.71 | -0.30 | 1.05 |
| 10 missing means |  | | -0.10 | 1.40 | -0.15 | 1.13 | -0.19 | 0.73 | -0.42 | 1.27 |
| **30 trials** | 0.02 | (-0.33 , 0.37) |  |  |  |  |  |  |  |  |
| 5 missing means |  | | 0.02 | 0.97 | -0.02 | 0.83 | -0.06 | 0.76 | -0.09 | 0.87 |
| 10 missing means |  | | 0.00 | 1.23 | -0.03 | 0.97 | -0.05 | 0.76 | -0.17 | 0.97 |
| 15 missing means |  | | 0.01 | 1.44 | -0.02 | 1.10 | 0.00 | 0.76 | -0.13 | 1.21 |

Results are given for trials with smaller sample sizes (average of 23 patients per trial) and allocation of the smallest trials to have missing mean values. Imprecision is the ratio of widths of confidence intervals for the intervention effect [width when estimating missing means : width when all means available]

**Table S12 GALA results: missing mean**

|  |  |  | **Method for missing mean replacement** | | | | | | | |
| --- | --- | --- | --- | --- | --- | --- | --- | --- | --- | --- |
|  | **Complete data result** | | **Hozo (ref)** | | **Bland (ref)** | | **Wan (ref)** | | **None, omit study** | |
|  | **Mean difference (days)** | **95% confidence interval** | **Bias** | **Imprecision** | **Bias** | **Imprecision** | **Bias** | **Imprecision** | **Bias** | **Imprecision** |
| **Scenario** |  |  |  |  |  |  |  |  |  |  |
| **5 trials** | 2.48 | (-0.12 , 5.08) |  |  |  |  |  |  |  |  |
| 2 missing means |  | | -2.69 | 0.33 | -2.61 | 0.33 | -2.44 | 0.33 | -2.22 | 0.42 |
| **10 trials** | 0.90 | (-0.25 , 2.05) |  |  |  |  |  |  |  |  |
| 2 missing means |  | | -0.27 | 1.09 | -0.76 | 0.67 | -0.94 | 0.31 | -0.89 | 0.33 |
| 5 missing means |  | | -0.79 | 1.36 | -0.97 | 0.50 | -0.87 | 0.31 | -0.91 | 0.51 |
| **20 trials** | 0.19 | (-0.23 , 0.61) |  |  |  |  |  |  |  |  |
| 5 missing means |  | | 0.97 | 2.67 | 0.19 | 1.67 | -0.22 | 0.73 | -0.17 | 0.90 |
| 10 missing means |  | | 0.83 | 3.15 | 0.15 | 1.93 | -0.18 | 0.73 | -0.14 | 1.02 |
| **30 trials** | 0.02 | (-0.33 , 0.37) |  |  |  |  |  |  |  |  |
| 5 missing means |  | | -0.01 | 1.09 | -0.02 | 0.87 | -0.02 | 0.76 | 0.06 | 0.87 |
| 10 missing means |  | | -0.02 | 2.21 | 0.03 | 1.43 | 0.01 | 0.76 | -0.05 | 1.23 |
| 15 missing means |  | | -0.22 | 2.47 | -0.08 | 1.61 | 0.00 | 0.76 | 0.09 | 1.66 |

Results are given for trials with smaller sample sizes (average of 23 patients per trial) and random allocation trials to have missing mean values. Imprecision is the ratio of widths of confidence intervals for the intervention effect [width when estimating missing means : width when all means available]

**Table S13 GALA results: missing mean**

|  |  |  | **Method for missing mean replacement** | | | | | | | |
| --- | --- | --- | --- | --- | --- | --- | --- | --- | --- | --- |
|  | **Complete data result** | | **Hozo (ref)** | | **Bland (ref)** | | **Wan (ref)** | | **None, omit study** | |
|  | **Mean difference (days)** | **95% confidence interval** | **Bias** | **Imprecision** | **Bias** | **Imprecision** | **Bias** | **Imprecision** | **Bias** | **Imprecision** |
| **Scenario** |  |  |  |  |  |  |  |  |  |  |
| **5 trials** | 2.48 | (-0.12 , 5.08) |  |  |  |  |  |  |  |  |
| 2 missing means |  | | -0.47 | 1.58 | -1.47 | 0.92 | -2.69 | 0.33 | -2.69 | 0.51 |
| **10 trials** | 0.90 | (-0.25 , 2.05) |  |  |  |  |  |  |  |  |
| 2 missing means |  | | -1.53 | 0.90 | -1.27 | 0.61 | -0.89 | 0.31 | -0.83 | 0.33 |
| 5 missing means |  | | 0.24 | 1.57 | -0.66 | 0.95 | -0.95 | 0.31 | -0.90 | 0.34 |
| **20 trials** | 0.19 | (-0.23 , 0.61) |  |  |  |  |  |  |  |  |
| 5 missing means |  | | 0.30 | 3.13 | -0.08 | 1.90 | -0.17 | 0.73 | -0.07 | 0.79 |
| 10 missing means |  | | 1.26 | 3.65 | 0.26 | 2.18 | -0.25 | 0.71 | -0.09 | 1.10 |
| **30 trials** | 0.02 | (-0.33 , 0.37) |  |  |  |  |  |  |  |  |
| 5 missing means |  | | 2.95 | 4.99 | 0.89 | 2.79 | -0.02 | 0.76 | 0.03 | 0.79 |
| 10 missing means |  | | 3.41 | 5.30 | 1.11 | 2.96 | -0.06 | 0.76 | -0.03 | 0.90 |
| 15 missing means |  | | 3.95 | 5.39 | 1.26 | 3.01 | -0.10 | 0.76 | 0.04 | 0.99 |

Results are given for trials with smaller sample sizes (average of 23 patients per trial) and allocation of the largest trials to have missing mean values. Imprecision is the ratio of widths of confidence intervals for the intervention effect [width when estimating missing means : width when all means available]

**Table S14 GALA results: missing mean**

|  |  |  | **Method for missing mean replacement** | | | | | | | |
| --- | --- | --- | --- | --- | --- | --- | --- | --- | --- | --- |
|  | **Complete data result** | | **Hozo (ref)** | | **Bland (ref)** | | **Wan (ref)** | | **None, omit study** | |
|  | **Mean difference (days)** | **95% confidence interval** | **Bias** | **Imprecision** | **Bias** | **Imprecision** | **Bias** | **Imprecision** | **Bias** | **Imprecision** |
| **Scenario** |  |  |  |  |  |  |  |  |  |  |
| **5 trials** | -0.09 | (-1.06, 0.87) |  |  |  |  |  |  |  |  |
| 2 missing means |  | | -0.03 | 0.89 | -0.01 | 0.89 | -0.08 | 0.89 | 0.34 | 1.41 |
| **10 trials** | -0.31 | (-0.99 , 0.38) |  |  |  |  |  |  |  |  |
| 2 missing means |  | | 0.29 | 0.53 | 0.35 | 0.53 | 0.31 | 0.53 | 0.14 | 0.73 |
| 5 missing means |  | | 0.32 | 0.68 | 0.34 | 0.64 | 0.34 | 0.53 | 0.27 | 1.45 |
| **20 trials** | -0.04 | (-0.42 , 0.35) |  |  |  |  |  |  |  |  |
| 5 missing means |  | | 0.07 | 1.42 | 0.05 | 1.16 | 0.03 | 0.78 | -0.07 | 1.14 |
| 10 missing means |  | | 0.13 | 1.53 | 0.08 | 1.23 | 0.04 | 0.79 | -0.19 | 1.39 |
| **30 trials** | -0.12 | (-0.42 , 0.18) |  |  |  |  |  |  |  |  |
| 5 missing means |  | | 0.16 | 1.13 | 0.12 | 0.97 | 0.08 | 0.88 | 0.05 | 1.02 |
| 10 missing means |  | | 0.14 | 1.43 | 0.11 | 1.13 | 0.09 | 0.88 | -0.03 | 1.13 |
| 15 missing means |  | | 0.15 | 1.68 | 0.12 | 1.28 | 0.14 | 0.88 | 0.01 | 1.42 |

Results are given for trials with larger sample sizes (average of 87 patients per trial) and allocation of the smallest trials to have missing mean values. Imprecision is the ratio of widths of confidence intervals for the intervention effect [width when estimating missing means : width when all means available]

**Table S15 GALA results: missing mean**

|  |  |  | **Method for missing mean replacement** | | | | | | | |
| --- | --- | --- | --- | --- | --- | --- | --- | --- | --- | --- |
|  | **Complete data result** | | **Hozo (ref)** | | **Bland (ref)** | | **Wan (ref)** | | **None, omit study** | |
|  | **Mean difference (days)** | **95% confidence interval** | **Bias** | **Imprecision** | **Bias** | **Imprecision** | **Bias** | **Imprecision** | **Bias** | **Imprecision** |
| **Scenario** |  |  |  |  |  |  |  |  |  |  |
| **5 trials** | -0.09 | (-1.06, 0.87) |  |  |  |  |  |  |  |  |
| 2 missing means |  | | -0.12 | 0.89 | -0.04 | 0.89 | 0.13 | 0.89 | 0.35 | 1.14 |
| **10 trials** | -0.31 | (-0.99 , 0.38) |  |  |  |  |  |  |  |  |
| 2 missing means |  | | 0.94 | 1.82 | 0.45 | 1.13 | 0.27 | 0.53 | 0.32 | 0.55 |
| 5 missing means |  | | 0.42 | 2.28 | 0.24 | 0.83 | 0.34 | 0.53 | 0.30 | 0.86 |
| **20 trials** | -0.04 | (-0.42 , 0.35) |  |  |  |  |  |  |  |  |
| 5 missing means |  | | 1.20 | 2.91 | 0.42 | 1.82 | 0.01 | 0.79 | 0.06 | 0.99 |
| 10 missing means |  | | 1.06 | 3.44 | 0.38 | 2.10 | 0.05 | 0.79 | 0.09 | 1.12 |
| **30 trials** | -0.12 | (-0.42 , 0.18) |  |  |  |  |  |  |  |  |
| 5 missing means |  | | 0.13 | 1.27 | 0.12 | 1.02 | 0.12 | 0.88 | 0.20 | 1.02 |
| 10 missing means |  | | 0.12 | 2.58 | 0.17 | 1.67 | 0.15 | 0.88 | 0.09 | 1.43 |
| 15 missing means |  | | -0.08 | 2.88 | 0.06 | 1.88 | 0.14 | 0.88 | 0.23 | 1.93 |

Results are given for trials with larger sample sizes (average of 87 patients per trial) and random allocation of trials to have missing mean values. Imprecision is the ratio of widths of confidence intervals for the intervention effect [width when estimating missing means : width when all means available]

**Table S16 GALA results: missing mean**

|  |  |  | **Method for missing mean replacement** | | | | | | | |
| --- | --- | --- | --- | --- | --- | --- | --- | --- | --- | --- |
|  | **Complete data result** | | **Hozo (ref)** | | **Bland (ref)** | | **Wan (ref)** | | **None, omit study** | |
|  | **Mean difference (days)** | **95% confidence interval** | **Bias** | **Imprecision** | **Bias** | **Imprecision** | **Bias** | **Imprecision** | **Bias** | **Imprecision** |
| **Scenario** |  |  |  |  |  |  |  |  |  |  |
| **5 trials** | -0.09 | (-1.06, 0.87) |  |  |  |  |  |  |  |  |
| 2 missing means |  | | 2.10 | 4.26 | 1.10 | 2.49 | -0.12 | 0.89 | -0.12 | 1.36 |
| **10 trials** | -0.31 | (-0.99 , 0.38) |  |  |  |  |  |  |  |  |
| 2 missing means |  | | 0.38 | 0.55 | -0.32 | 1.51 | -0.06 | 1.03 | 0.32 | 0.52 |
| 5 missing means |  | | 1.45 | 2.64 | 0.55 | 1.60 | 0.26 | 0.52 | 0.31 | 0.57 |
| **20 trials** | -0.04 | (-0.42 , 0.35) |  |  |  |  |  |  |  |  |
| 5 missing means |  | | 0.53 | 3.42 | 0.15 | 2.08 | 0.06 | 0.79 | 0.16 | 0.86 |
| 10 missing means |  | | 1.49 | 3.99 | 0.49 | 2.38 | -0.02 | 0.78 | 0.14 | 1.19 |
| **30 trials** | -0.12 | (-0.42 , 0.18) |  |  |  |  |  |  |  |  |
| 5 missing means |  | | 3.09 | 5.82 | 1.03 | 3.25 | 0.12 | 0.88 | 0.17 | 0.92 |
| 10 missing means |  | | 3.55 | 6.18 | 1.25 | 3.45 | 0.08 | 0.88 | 0.11 | 1.05 |
| 15 missing means |  | | 4.09 | 6.28 | 1.40 | 3.52 | 0.04 | 0.88 | 0.18 | 1.15 |

Results are given for trials with larger sample sizes (average of 87 patients per trial) and allocation of the largest trials to have missing mean values. Imprecision is the ratio of widths of confidence intervals for the intervention effect [width when estimating missing means : width when all means available]
